# HIV matters when diagnosing TB in young children: an ancillary analysis in children enrolled in the INPUT stepped wedge cluster randomized study

**DOI:** 10.1186/s12879-023-08216-w

**Published:** 2023-04-17

**Authors:** L Powell, L Denoeud-Ndam, N Herrera, R Masaba, B Tchounga, S Siamba, M Ouma, SJ Petnga, R Machekano, B Pamen, G Okomo, L Simo, M Casenghi, N Rakhmanina, A Tiam

**Affiliations:** 1 Division of Pediatric Infectious Diseases, Department of Pediatrics, Medical University of South Carolina, Charleston, SC USA; 2 Elizabeth Glaser Pediatric AIDS Foundation, Geneva, Switzerland; 3 Elizabeth Glaser Pediatric AIDS Foundation, Washington, DC USA; 4 Elizabeth Glaser Pediatric AIDS Foundation, Nairobi, Kenya; 5 Elizabeth Glaser Pediatric AIDS Foundation, Yaounde, Cameroon; 6 Department of Disease, Epidemic and pandemic control, Ministry of Health, Yaounde, Cameroon; 7 Department of Health, Homa Bay county Government, Homa Bay, Kenya

**Keywords:** Tuberculosis, HIV, Pediatrics, Malnutrition, Case finding

## Abstract

**Background:**

Children under age five years, particularly those living with HIV (CLHIV), are at risk for rapid progression of tuberculosis (TB). We aimed to describe TB clinical presentations, diagnostic pathways and treatment outcomes in CLHIV compared to children without HIV in Cameroon and Kenya.

**Methods:**

This sub-analysis of a cluster-randomized trial evaluating the integration of pediatric TB services from May 2019 to March 2021 enrolled children age < 5 years with TB. We estimated the HIV infection rate with 95% confidence interval (CI). We compared TB clinical presentations, diagnostic pathways and treatment outcomes in CLHIV and children without HIV. Finally, we investigated whether HIV infection was associated with a shorter time to TB diagnosis (≤ 3 months from symptoms onset) after adjusting for covariates. Univariable and multivariable logistic regression analysis were performed with adjusted odds ratios (AORs) presented as measures of the association of covariates with HIV status and with shorter time to TB diagnosis.

**Results:**

We enrolled 157 children with TB (mean age was 1.5 years) and 22/157 (14.0% [9.0-20.4%]) were co-infected with HIV. CLHIV were more likely to initially present with acute malnutrition (AOR 3.16 [1.14–8.71], p = 0.027). Most TB diagnoses (140/157, 89%) were made clinically with pulmonary TB being the most common presentation; however, there was weak evidence of more frequent bacteriologic confirmation of TB in CLHIV, 18% vs. 9% (p = 0.067), due to the contribution of lateral-flow urine lipoarabinomannan to the diagnosis. HIV positivity (AOR: 6.10 [1.32–28.17], p = 0.021) was independently associated with a shorter time to TB diagnosis as well as fatigue (AOR: 6.58 [2.28–18.96], p = 0.0005), and existence of a household contact diagnosed with TB (AOR: 5.60 [1.58–19.83], p = 0.0075), whereas older age (AOR: 0.35 [0.15–0.85], p = 0.020 for age 2–5 years), night sweats (AOR: 0.24 [0.10–0.60], p = 0.0022) and acute malnutrition (AOR: 0.36 [0.14–0.92], p = 0.034) were associated with a delayed diagnosis. The case fatality rate was 9% (2/22) in CLHIV and 4% (6/135) in children without HIV, p = 0.31.

**Conclusions:**

These results altogether advocate for better integration of TB services into all pediatric entry points with a special focus on nutrition services, and illustrate the importance of non-sputum-based TB diagnostics especially in CLHIV.

**Trial registration:**

NCT03862261, first registration 05/03/2019.

**Supplementary Information:**

The online version contains supplementary material available at 10.1186/s12879-023-08216-w.

## Background

Tuberculosis is one of the top ten causes of death in children worldwide [1]. In 2020, children accounted for 11% (1.1 million) of global TB cases [2]. Mathematical models predict half the cases are in those less than five years of age [3] and approximately 10% (~ 21,000) of total deaths from TB-HIV co-infection occur in children [2]. The coronavirus 2019 (COVID-19) pandemic has led to a catastrophic decline in the number of cases newly diagnosed and reported with TB as well as a notable increase in TB deaths, including in those infected with HIV, due to the reduced availability of TB diagnostic and treatment services [2]. Pre-pandemic trends showed that the biggest case detection gap was in children less than five years, with 69% missed diagnoses (under-diagnosed and under-reported) [4]. Despite the scale up of antiretroviral therapy (ART), children living with HIV are still at a higher risk of contracting TB than the general population [5, 6].

In children, the laboratory-based diagnosis of TB is particularly challenging due to the paucibacillary nature of the disease and the difficulty to obtain respiratory samples for bacteriological confirmation [7]. The diagnosis of TB in CLHIV is further complicated by their immunocompromised status, as current available diagnostic assays are characterized by a decreased sensitivity in this population [8, 9]. CLHIV have non-specific clinical presentations of TB, similar to all children [10], with clinical and radiologic features overlapping with other HIV-related opportunistic infections in this population [11].

Major advances in the diagnosis of TB in the last decade include highly specific, semi-automated nucleic acid amplification tests, Cepheid Xpert® MTB/RIF test and Xpert® MTB/RIF Ultra test, (Cepheid, USA; referred to as Xpert and Xpert Ultra) to detect *Mycobacterium tuberculosis* and provide information on rifampin resistance [12, 13]. Xpert Ultra, due to its lower limit of detection, has superior sensitivity compared to Xpert for those with smear-negative TB [14], which is more common in children and those living with HIV. In a recent Cochrane Review on the impact of Xpert on TB outcomes in patients, it was determined that Xpert likely leads to a reduction in mortality in those known to be infected with HIV (OR: 0.80 (95%CI 0.67–0.96) [15]. A more recent diagnostic assay, the Determine™ TB-LAM Ag, lateral flow urine lipoarabinomannan (LAM) antigen (Ag) assay (Abbott, USA; referred to as LF-LAM), used for the detection of LAM-Ag in the urine of individuals with TB, has also shown improved sensitivity among individuals co-infected with HIV compared to the general population [16]. In addition a positive LAM-Ag assay may be a risk factor of mortality for this special population [17].

Due to this evidence, the World Health Organization (WHO) recommends Xpert and Xpert Ultra as the initial diagnostic test for TB and rifampin resistance in children, and further recommends the use of LF-LAM to assist in the diagnosis of TB in CLHIV [18].

The objectives of this study were (i) to determine the rate of HIV infection among children under age five with presumptive and confirmed pediatric TB diagnosis; (ii) to describe the characteristics of children with HIV in terms of clinical presentations, pathways to TB diagnosis and TB treatment outcomes, and (iii) to compare them to those of children not living with HIV. As HIV status was associated with a shorter time from symptom onset to TB diagnosis in preliminary analysis, we assessed which other co-variates also demonstrated an association with a shorter time to diagnosis. We then confirmed if the effect of HIV infection remained in multivariate analysis.

## Methods

### Study design and setting

This is a sub-analysis of the INPUT study. The INPUT study is a multi-national, cluster-randomized, stepped-wedge trial, which evaluates the major components of the Catalyzing Pediatric TB (CaP-TB) project, integrating pediatric TB screening and diagnostics into non-TB child healthcare services at the hospital and primary health care level. The study was conducted in six district-level hospitals and their attached primary health care centers in Cameroon and Kenya for a total of 32 facilities. The details of this protocol have been previously published and include a detailed summary of the standard of care for TB management in Kenya and Cameroon, and the CaP-TB package of interventions [19].

### Study population

From May 2019 to March 2021 (with a pause due to COVID-19 from April to July 2020), the INPUT study prospectively enrolled young children under the age of five years with a presumptive diagnosis of TB based on TB signs and symptoms, and followed them through TB diagnosis investigations and treatment where applicable. In this ancillary analysis, the study population included all INPUT study participants who were confirmed with TB, bacteriologically or clinically. This population comprised of both CLHIV and children living without HIV, all with TB.

### Data collection

In the INPUT study, an electronic data capture system developed by CliniOps (Fremont, CA, USA) was used to standardize data collection across the study sites and to compile the data into one unified dataset. Research nurses entered data directly into a tablet via an electronic case report form. Data were synced regularly through a secured server, with real-time access of the consolidated data across all sites.

From the main INPUT study database, we extracted baseline socio-demographic data such as country, service entry point, age, sex, and education level of the caregiver. We also extracted clinical information: HIV maternal and child status, moderate or severe acute malnutrition, TB contact in the household, signs and symptoms of TB and their date of onset, type of TB investigations performed (specimen collected, assay used, radiologic testing performed and result), TB classification (pulmonary TB [PTB] or extrapulmonary TB [EPTB]), and type of TB diagnosis (bacteriologic or clinical). Children with bacteriologically confirmed TB were those from whom a biological specimen (i.e. sputum (expectorated or induced), gastric aspirate, nasopharyngeal aspirate, lymph node aspirate, cerebrospinal fluid, urine) was positive by smear microscopy, culture or WHO approved rapid diagnostics such as Xpert or LF-LAM. Children with clinically diagnosed TB were those who, based on symptoms, history, and sometimes radiologic abnormalities, had been diagnosed with active TB by a medical practitioner and received a full course of TB treatment.

### Statistical analysis

The prevalence of HIV coinfection among children presumed and diagnosed with TB was estimated together with the associated 95% confidence interval (CI). Demographic and clinical characteristics of children diagnosed with TB at enrollment were summarized using means (standard deviations) or medians (interquartile range) for continuous variables depending on distribution, and proportions for categorical variables, stratified by HIV status. To compare the characteristics between CLHIV and children without HIV (either HIV-negative or unknown status), Chi Square or Fisher’s exact tests of independence were used for categorical variables, whereas a Wilcoxon-Mann-Whitney test was used for continuous variables. Univariable and multivariable logistic regression was performed to identify the demographics and clinical presentations associated with HIV status, and with a reduced time to TB diagnosis. This latter analysis was performed because we noticed that HIV infection was associated with a shorter time to TB diagnosis and we felt important to measure this association after adjustment for other co-variates. Time to TB diagnosis was defined as the time from the month of onset of TB signs and symptoms to the month of TB diagnosis and was dichotomized to equal or less than 3 months versus greater than 3 months. Variables which had a p-value less than 0.2 in univariable analysis were then included in the multivariable model. A backwards method allowed retention of variables in the models with a significance of 0.05, while country and age (dichotomized; 2 to 5 years versus younger than 2 years) were forced in the models. Relevant interaction terms were also tested. Analysis was performed using SAS (Cary, NC, USA), Version 9.4.

We finally compared TB treatment outcomes among CLHIV and children without HIV, using the WHO standard definition of treatment outcome assessed two months after treatment completion [20].

### Power and sample size

No a priori power or sample size estimates were performed for this exploratory sub-analysis. We used the sample that was available from the INPUT study.

## Results

### Baseline characteristics and prevalence of TB-HIV co-infection

Of the 790 children enrolled in the INPUT study with TB signs and symptoms who were investigated for TB (*See Additional file 1*), 53/790, 6.7% (95%CI [5.1-8.7%]) were CLHIV. After TB investigations, one fifth (157/790, 20%) of the overall cohort was diagnosed with TB, they are the focus of this sub-analysis. A total of 37/157 (24%) participants were from Cameroon and 120/157 (76%) participants were from Kenya, with most children living in urban communities (115/157, 73%) *(*Table 1). Among children diagnosed with TB, 127/157 (81%) had a negative HIV status and 8/157 (5%) had an unknown status (5 born from HIV-negative mothers, one from an HIV-positive mother, and two from mothers with unknown HIV status). Overall, HIV prevalence among children diagnosed with TB was 22/157, 14.0% (95%CI [9.0-20.4%]).

**Table 1:** Baseline characteristics according to HIV status

	HIV-negative/unknown n (%)	HIV-positive n (%)	All n (%)	p-value (Chi -Square value)
Total	135	22	157	
*Socio-demographic characteristics*				
Age (years)				
< 2	87 (64.4)	9 (40.9)	96 (61.2)	0.036
2–5	48 (35.6)	13 (59.1)	61 (38.8)	
Gender				0.48
Male	75 (55.6)	14 (63.6)	89 (56.7)	
Female	60 (44.4)	8 (36.4)	68 (43.3)	
Country of residence				0.13
Cameroon	29 (21.5)	8 (36.4)	37 (23.6)	
Kenya	106 (78.5)	14 (63.6)	120 (76.4)	
Place of residence				0.27
Rural	34 (25.2)	8 (36.4)	42 (26.8)	
Urban	101 (74.8)	14 (63.6)	115 (73.2)	
Caregiver highest level of education^1^				0.0020
never attended/unknown	43 (31.9)	0 (0)	43 (27.4)	
primary/upper level	92 (68.1)	22 (100)	114 (72.6)	
Maternal HIV status				< 0.0001
Positive	10 (8.1)	19 (86.4)	29 (19.9)	
Negative	96 (77.4)	0 (0)	96 (65.7)	
Unknown	18 (14.5)	3 (13.6)	21 (14.4)	
Service^2^				< 0.0001
MCH/PMTCT	5 (3.7)	0 (0)	5 (3.2)	
Outpatient department	62 (45.9)	4 (18.2)	66 (42.0)	
Nutrition rehabilitation center	4 (3.0)	0 (0)	4 (2.6)	
HIV/CCC	2 (1.5)	11 (50.0)	13 (8.3)	
Inpatient department	25 (18.5)	3 (13.6)	28 (17.8)	
Chest clinic/TB room	24 (17.8)	2 (9.1)	26 (16.6)	
Emergency room	2 (1.5)	1 (4.5)	3 (1.9)	
Other	11 (8.1)	1 (4.5)	12 (7.6)	
*Clinical presentation*				
Cough	105 (77.8)	21 (95.5)	126 (80.3)	0.053
Fever	74 (54.8)	15 (68.2)	89 (56.7)	0.24
Night sweats	57 (42.2)	10 (45.5)	67 (42.7)	0.78
Appetite loss/failure to thrive	54 (40.3)	13 (59.1)	67 (42.7)	0.093
Fatigue/reduced playfulness/decreased activity	53 (39.9)	11 (50.0)	65 (41.4)	0.34
Dyspnea	20 (14.8)	4 (18.2)	24 (15.3)	0.68
Wheeze	14 (10.5)	2 (9.1)	16 (10.2)	0.85
Abnormal pulmonary auscultation/percussion	31 (23.1)	9 (40.9)	40 (25.5)	0.073
Adenitis	24 (17.9)	3 (13.6)	27 (17.2)	0.63
Edema	7 (5.2)	2 (9.1)	9 (5.7)	0.47
Dehydration	5 (3.7)	2 (9.1)	7 (4.5)	0.26
Pallor	7 (5.2)	3 (13.6)	10 (6.4)	0.13
Pruritus	1 (0.8)	1 (4.6)	2 (1.3)	0.16
Moderate acute malnutrition^3^	18 (13.3)	3 (13.6)	21 (13.4)	0.97
Severe acute malnutrition^3^	21 (15.6)	7 (31.8)	28 (17.8)	0.065
*Contact history*				
Someone coughing in household	49 (37.1)	8 (36.4)	57 (37.0)	0.95
Household contact diagnosed with TB	29 (21.5)	3 (13.6)	32 (20.7)	0.40
Household contact on TB treatment	25 (18.5)	2 (9.1)	27 (17.2)	0.28

^1^Education: primary = primary, junior high school, senior high school; upper = beyond high school

^2^Service: MCH/PMTCT = maternal and child health care, prevention of mother to child transmission; HIV/CCC = HIV/comprehensive care center

^3^The presence of moderate and severe acute malnutrition was evaluated routinely by health care workers at the site and this information was abstracted by the study team. We did not record weight-for-age Z-score or mid-upper arm circumference

Among children with TB, 89/157 (57%) were male and the mean age was 1.5 years with 61% (n = 96) under the age of 2 years. CLHIV were significantly older than those without HIV (59% vs. 36%; p = 0.036). *(*Table 1*).* Overall 50% (n = 11) CLHIV were screened for TB in the HIV clinic while the main entry point for children without HIV was the outpatient department (46%, n = 62). The proportion of children enrolled from the inpatient department was 18% (n = 28) and was similar in both groups.

### Clinical presentations

Respiratory symptoms, which consisted of dyspnea, cough, rhinitis, or cold, were reported as the reason for coming to pediatric entry points in 129/157 (85%) children. Cough was the most common symptom in both groups of CLHIV and children without HIV, followed by fever, night sweats, and appetite loss or failure to thrive (Table 1).

Overall, 57/157 (37%) had someone coughing in their household, they were 8/22 (36%) among CLHIV and 49/135 (37%) among children without HIV; 27/153 (17%) of children had a household contact on TB treatment *(*Table 1*).*

In univariate analysis, there was no evidence of difference in the clinical characteristics in the two groups. Variables which had a p value of < 0.2 and were included in the first step of the multivariable analysis to look for an association with HIV status were: cough (OR = 6.00 [0.78–46.45], p = 0.086)), loss of appetite or failure to thrive (OR = 2.14 [0.86–5.36], p = 0.10), abnormal pulmonary auscultation (OR = 2.30 [0.90–5.89], p = 0.082), pallor (OR = 2.87 [0.68–12.04], p = 0.15), pruritus (OR = 6.33 [0.38-105.17], p = 0.19) and moderate or severe acute malnutrition (OR = 2.05 [0.82–5.14], p = 0.13) *(*Table 2).

**Table 2:** Univariate and multivariate logistic regression analysis of signs and symptoms associated with a positive HIV status

Variable	Univariate logistic regression		Multivariate logistic regression ^2^	
	Unadjusted OR (95% CI)	p-value (Wald test)	Adjusted OR (95% CI)	p-value (Wald test)
*Demographics*				
Age (years)^1^		0.040		0.019
< 2 (ref)	1		1	
2–5	2.62 (1.04–6.60)		3.24 (1.22–8.63)	
Country^1^				
Kenya (ref)	1		1	
Cameroon	2.09 (0.80–5.46)		2.52 (0.90–7.02)	
*Clinical Presentation*				
Cough^1^	6.00 (0.78–46.45)	0.086		
Fever	1.77 (0.68–4.61)	0.25		
Night sweats	1.14 (0.46–2.82)	0.78		
Appetite loss/failure to thrive^1^	2.14 (0.86–5.36)	0.10		
Fatigue/reduced playfulness/decreased activity	1.51 (0.61–3.73)	0.37		
Dyspnea	1.28 (0.39–4.17)	0.69		
Wheeze	0.86 (0.18–4.06)	0.85		
Abnormal pulmonary auscultation/percussion^1^	2.30 (0.90–5.89)	0.082		
Adenitis	0.72 (0.20–2.64)	0.63		
Edema	1.81 (0.35–9.36)	0.48		
Dehydration	2.58 (0.47–14.21)	0.28		
Pallor^1^	2.87 (0.68–12.04)	0.15		
Pruritus^1^	6.33 (0.38-105.17)	0.20		
Jaundice	-	-		
Moderate or severe acute malnutrition^1^	2.05 (0.82–5.14)	0.13	3.16 (1.14–8.71)	0.027

^1^ Included in multivariate model at p < 0.2. Country and age were forced in the multivariate model

^2^ The final multivariate model retains variables with p < 0.05

OR odds ratio, Wald estimate

CI confidence interval

Malnutrition (adjusted odds ratio (AOR): 3.16 [1.14–8.71], p = 0.027) and older age (AOR: 3.24 [1.22–8.63], p = 0.019) were independently associated with a positive HIV status (Table 2*).*

### Diagnostic pathways

Overall, a biologic specimen was collected for TB diagnosis in 96/157 (61%) of children (n = 96). Gastric aspirate was the main specimen type collected (Table 3). Xpert was performed in 65/135 (48%) of children without HIV with 12/65 (19%) returning positive (specimens included: one sputum, three nasopharyngeal aspirate, seven gastric aspirates, and one lymph node). In CLHIV, 12/22 (55%) had an Xpert assay performed with 1/12 (8%) returning positive. Additionally, four CLHIV had a LF-LAM test performed on urine that returned positive.

**Table 3:** TB diagnostic investigations according to HIV status

	HIV-negative/unknown n (%)	HIV-positive n (%)	All n (%)	p-value (Fisher exact test)
Total	135	22	157	
Specimen collected	79 (58.5%)	17 (77.3%)	96 (61.1%)	0.10
Specimen type				0.0070
Sputum	11 (13.9)	1 (5.9)	12 (12.5)	
Induced sputum	4 (5.1)	0 (0)	4 (4.2)	
Lymph node aspirate	4 (5.1)	0 (0)	4 (4.2)	
Cerebrospinal fluid	0 (0)	0(0)	0(0)	
Nasopharyngeal aspirate	8 (10.1)	2 (11.8)	10 (10.4)	
Gastric aspirate	52 (65.8)	10 (58.8)	62 (64.6)	
Urine^1^		4 (23.5%)	4 (4.2)	
Xpert assay (+ Ultra) performed	65 (48.1)	12 (54.5)	77 (49.0)	0.65
Xpert assay result				0.68
Positive	12 (18.8)	1 (8.3)	13 (17.1)	
Negative	52 (81.2)	11 (91.7)	63 (82.9)	
Smear microscopy performed	22(16.3)	5 (22.7)	27	0.54
Smear microscopy result				1.00
Positive	0 (0)	0(0)	0(0)	
Negative	21 (95.5)	5 (100)	26 (96.3)	
Indeterminate	1 (4.5)	0(0)	1 (3.7)	
Xray performed	85 (63.0)	12 (54.5)	97 (61.8)	0.48
Xray result				0.62
Normal	5 (5.9)	1 (8.3)	6 (6.2)	
Abnormal, consistent with TB	79 (92.9)	11 (91.7)	90 (93.2)	
Unknown	1 (1.2)	0 (0)	1 (1.0)	
TB diagnosis				0.067
Bacteriologic confirmation	12 (8.9)	5 (22.7)	17 (10.8)	
Clinical diagnosis	123 (91.1)	17 (77.3)	140 (89.2)	
TB Classification				0.41
pulmonary	104 (77.0)	19 (86.4)	123 (78.3)	
Extrapulmonary	31 (23.0)	3 (13.6)	34 (21.7)	
Types of extra-pulmonary TB				0.25
Lymph node	29	2	31	
Disseminated	1	1	2	
Osteo-articular	1	0	1	
Time from Symptom to Diagnosis				0.080
≤ 3 Months	86 (65.6)	19 (86.4)	105 (68.6)	
> 3 Months	45 (34.4)	3 (13.6)	48 (31.4)	

^1^ Urine was collected for LF-LAM test

Most TB cases (140/157, 89%) were diagnosed clinically, however there is weak evidence of more frequent bacteriologic confirmation of TB among CLHIV (5/22, 23%) compared to children without HIV (12/135, 9%), p = 0.067.

PTB was the most frequent presentation of TB in both CLHIV and children without HIV, 19/22 (86%) and 104/135 (77%), respectively. Of the 34/157 (22%) EPTB, 31 had lymph node TB (two were CLHIV), 2 had disseminated TB (one was a CLHIV) and 1 had osteo-articular TB (a child living without HIV).

Overall, 105/157 (69%) of TB cases were diagnosed within 3 months after the onset of TB symptoms, 19/22 (86%) in CLHIV and 86/135 (66%) in children living without HIV, p = 0.08 *(*Table 3*).*

In univariable analysis of the factors associated with a shorter time to TB diagnosis, together with HIV positivity, the presence of cough, night sweats, fatigue, abnormal pulmonary auscultation, acute malnutrition, and existence of a contact coughing or being diagnosed with TB in the household carried significance at a level of 0.2. Thus, these covariates were included in the first step of multivariable modelling along with age, country and HIV status (Table 4*).* In the final multivariate model, HIV positivity (AOR: 6.10 [1.32–28.17]), fatigue (OR: 6.58 [2.28–18.96]), and existence of a household contact diagnosed with TB (AOR: 5.60 [1.58–19.83]) were independently associated with a shorter time while older age (AOR: 0.35 [0.15–0.85]), night sweats (OR: 0.24 [0.10–0.60]) and malnutrition (AOR: 0.36 [0.14–0.92]) were associated with a longer time to TB diagnosis; p < 0.05.

**Table 4:** Univariate and multivariate logistic regression of the factors associated with a reduced time to TB diagnosis (time from onset of TB signs and symptoms to TB diagnosis ≤ 3 months)

Variable	Univariate Logistic Regression		Multivariate Logistic Regression^2^	
*Demographics*	Unadjusted OR (95%CI)	p-value (Wald test)	Adjusted OR (95%CI)	p-value (Wald test)
Age (years)^1^		0.32		0.020
< 2 (ref)^1^	1		1	
2–5	0.70 (0.35–1.40)		0.35 (0.15–0.85)	
Kenya (ref)	1		1	
Cameroon	7.18 (2.08–24.78)		5.10 (1.29–20.24)	
*HIV status*				
HIV negative or unknown (ref)	1		1	
HIV positive^1^	3.31 (0.93–11.79)	0.064	6.10 (1.32–28.17)	0.021
*Clinical Presentation*				
Cough^1^	2.07 (0.90–4.74)	0.089		
Fever	1.22 (0.61–2.43)	0.57		
Night sweats^1^	0.50 (0.25–0.99)	0.049	0.24 (0.10–0.60)	0.0022
Appetite loss/failure to thrive	0.74 (0.37–1.47)	0.38		
Fatigue/reduced playfulness/decreased activity^1^	3.30 (1.52–7.17)	0.0026	6.58 (2.28–18.96)	0.0005
Dyspnea	1.13 (0.44–2.94)	0.80		
Wheeze	0.56 (0.19–1.59)	0.27		
Abnormal pulmonary auscultation/percussion^1^	2.22 (0.94–5.28)	0.071		
Adenitis	0.85 (0.35–2.07)	0.72		
Edema	1.16 (0.22–6.21)	0.86		
Dehydration	1.16 (0.22–6.21)	0.86		
Pallor^1^	4.45 (0.55–36.19)	0.16		
Pruritus	-	-		
Moderate or severe acute malnutrition^1^	0.61 (0.30–1.25)	0.18	0.36 (0.14–0.92)	0.034
*TB characteristics*				
Bacteriologically confirmed TB	2.31 (0.63–8.44)	0.21		
Someone coughing in household^1^	1.92 (0.89–4.13)	0.095		
Household contact diagnosed with TB^1^	2.69 (0.96–7.52)	0.060	5.60 (1.58–19.83)	0.0075

^1^retained in the first step of multivariate model at 0.2

^2^ The final model retained variables with p < 0.05

CLHIV had more frequent malnutrition, and because HIV status and malnutrition had reverse effects on diagnostic delay (the first being associated with a shorter time to diagnosis, the second with a longer time), we further tested the interaction between them. It was found to not be significant. In other words, the effect of malnutrition on delayed TB diagnosis was similar in CLHIV and children without HIV.

### Treatment outcomes

Two months after TB treatment completion, 10 children were classified as cured and 126 as having completed treatment (altogether 136/157, 87% with a favorable treatment outcome as defined by the WHO [20]) (Table 5). Among CLHIV, 82% (n = 18) had a favorable treatment outcome, versus 87% (n = 118) in those living without HIV, p = 0.50. Lastly, a total of 8 (5.1%) deaths were observed; the case fatality rate was 9.1% (n = 2) among CLHIV compared to 4.4% (n = 6) among those living without HIV; p = 0.31.

**Table 5:** TB treatment outcomes

	HIV-negative/unknown n (%)	HIV-positive n (%)	All n (%)
Total	135	22	157
WHO classification of treatment outcomes			
Cured	9 (6.7)	1 (4.5)	10 (6.4)
Treatment completed	109 (80.7)	17 (77.3)	126
Treatment failed	0	0	0
Died	6 (4.4)	2 (9.1)	8 (5.1)
Lost to follow-up	7 (5.2)	1 (4.5)	8 (5.1)
Not evaluated^1^	4 (3.0)	1 (4.5)	5
WHO favorable treatment outcome^2^	118 (87.4)	18 (81.8)	136 (86.6)

^1^Reasons include: participant relocated n = 3 (including one CLHIV), treatment not completed n = 2

^2^Defined by WHO as cured or treatment completed

## Discussion

The overall prevalence of TB-HIV co-infection was 14% among children under age five years diagnosed with TB in our study. Studies from Cameroon and Kenya have found estimates in children less than five years of age with TB-HIV co-infection to be between 41 and 52% [21, 22]. The prevalence of co-infection was found to be lower than in the previously mentioned studies; however, the proportion of CLHIV among those who were diagnosed with TB was found to be higher than in children who had presumed but were not diagnosed with TB in the INPUT study. This is consistent with previous studies showing that CLHIV have a higher risk of TB disease than the general population [23, 24]. The COVID-19 pandemic has worsened the TB case detection gap [2], with only half of the 1.1 million children (with half under the age of 5 [3]) becoming ill with TB each year currently being reported [4]. This demonstrates the importance of TB screening for young children including CLHIV, not only at HIV entry points, but also integrated into other entry points where ill children are seen.

In this study, CLHIV did not necessarily have a wider variety of clinical presentations, but they did present with acute malnutrition more frequently compared to those living without HIV. This exploratory analysis had limited power and did not determine a statistically significant difference in the case-fatality rate among the two populations. In published studies, TB manifestations have been shown to be more severe and progression to death more rapid among CLHIV compared to those living without HIV, with overall high rates of mortality, specifically amongCLHIV under the age five years [24] and in those with poor nutritional status [25, 26].

The importance of considering malnutrition as a key element in TB case finding, especially among CLHIV, is highlighted by two findings in this study. The first is the significant association found between HIV infection and acute malnutrition among children diagnosed with TB. The second is that malnutrition was independently associated with a significant delay in TB diagnosis, both in HIV-infected and uninfected populations. Delayed TB diagnosis and treatment initiation is associated with higher disease transmission [27], and higher disease severity and mortality [28]. Several studies have found that HIV increases the time to TB diagnosis, with examples in adults in Guinea-Bissau [29] and in children in Ethiopia [30]. In this study, we found the contrary, CLHIV were diagnosed more rapidly than children without HIV. However, malnutrition delayed TB diagnosis and was more frequent in CLHIV. Delays in TB diagnosis are a result of both patient delays in seeking care as well as health system delays in diagnosing children who come to healthcare facilities [31]. In this study, it is unclear whether HIV and malnutrition played a predominant role on patient delays or health system delays. It is possible that caregivers of CLHIV may be more inclined to come early to facilities when their child is ill. Clinicians caring for CLHIV should be more aware of TB signs and symptoms in this population and perform more systematic TB screening as part of routine HIV care, including providing TB preventive therapy. In CLHIV, the use of LF-LAM and observed trend towards more frequent bacteriologic confirmation might also have contributed to a more rapid TB diagnosis. Malnutrition may be a consequence rather than a cause of delay in TB diagnosis as undernutrition is not only a risk factor for TB [32], but TB can lead to malnutrition [33]. Malnutrition may also be a marker of unfavorable socio-economic condition of the family associated with more difficult access to quality health care. Finally, it is possible that children with malnutrition were not accurately screened for TB signs and symptoms in nutrition entry points as studies have shown in India [34]. This altogether stresses the importance of better integration of TB screening, as well as HIV screening, in nutrition entry points and into health care packages for children.

The other determinants of time from symptom onset to TB diagnosis were fatigue and existence of a household contact diagnosed with TB, which reduced the time to diagnosis, while night sweats and older age increased the time to diagnosis. Having a household contact with TB was associated with a faster TB diagnosis, which may reflect efficient contact tracing in these households. Fatigue and younger age may have been significant prompting caregivers to bring their child to healthcare facilities more rapidly. We are not able to explain why children with night sweats were diagnosed significantly later than other children.

Although most cases were diagnosed clinically, CLHIV tended to have more bacteriologic confirmations compared to children living without HIV. Obtaining sputum in children, especially those under the age of five years, irrespective of HIV status, is difficult and it is clear from our results that LF-LAM diagnostic test performed on urine samples contributed a lot to bacteriologic confirmation among CLHIV. This sub-analysis highlights and stresses the importance of the newly updated WHO recommendations on the use of currently commercially available LF-LAM to aid in the diagnosis of TB among the people living with HIV [18].

Rapid, non-sputum-based diagnostic tools such as urine LF-LAM, urine Xpert Ultra or stool Xpert have shown value for TB diagnosis in people living with HIV and in the general pediatric population including the outpatient setting [35, 36].

Limitations of our study included the small number of CLHIV which limited our ability to draw generalizable conclusions, and the number of children with an unknown HIV status. Among fifteen children presenting at enrollment with an unknown status, a HIV test was performed in seven by the time of TB diagnosis, and was found to be negative. The eight children with remaining unknown HIV status were grouped with the cohort of children living without HIV, which may have contributed to a decreased co-infection rate. Among them, one was born from an HIV-positive mother. This child was in very critical condition at baseline and died soon after TB diagnosis was made, but before HIV testing. The seven other children were born from HIV-negative (n = 5) or unknown (n = 2) mothers, making it less likely that they would have been HIV-positive.

Because we did not collect the weight-for-age z-score or mid-upper arm circumference but only determined if moderate or severe acute malnutrition was present (as per indicated in facility records), we were not able to assess if the classification of children with moderate and severe malnutrition was accurate based on standardized definitions. This potential inaccuracy is the reason why we decided to group children with reported moderate and acute severe malnutrition together in our analyses.

Despite these limitations, this study gives important insights into specific clinical presentations and pathways to diagnosis of TB in CLHIV.

## Conclusions

In this sub-analysis of the INPUT study comparing children under age five years with TB-HIV co-infection to those with TB living without HIV in Kenya and Cameroon, we identified a lower-than-expected proportion of TB-HIV co-infection. Children with TB and HIV presented with malnutrition more frequently compared to those living without HIV. The use of LF-LAM in CLHIV contributed to bacteriologic confirmation of TB in these children. HIV positivity was significantly associated with a shorter time to TB diagnosis; however, children with malnutrition were diagnosed less rapidly. This altogether advocates for the need for better integration of TB services (including screening efforts and contact investigation) into all pediatric care entry points with a special focus on nutrition services, and for the importance of non-sputum-based TB diagnostics, especially in children living with HIV, to aid in more rapid diagnosis of TB.

## Electronic supplementary material

Below is the link to the electronic supplementary material.

Supplementary Material 1

## Data Availability

The datasets used and/or analyzed during the current study are available from the corresponding author on reasonable request.

## Abbreviations

Ag: Antigen
AOR: Adjusted odds ratio
ART: Antiretroviral therapy
CaP-TB: Catalyzing pediatric TB innovations
CCC: Comprehensive care center for HIV
CLHIV: Children living with HIV
CNERSH: Cameroon national ethics committee for research in human health
CI: Confidence interval
COVID-19: Coronavirus disease 2019
EGPAF: Elizabeth Glaser Pediatric AIDS Foundation
EPTB: Extra-pulmonary TB
ERC: Ethics review committee
HIV: Human immunodeficiency virus
INPUT: Integrating Pediatric TB services into child healthcare services in Africa stepped-wedge cluster randomized study
IRB: Institutional Review Board
KNH UON: Kenyatta National Hospital-University of Nairobi
LF-LAM: Determine™ TB lateral flow urine lipoarabinomannan antigen assay
MCH: Maternal and Child Healthcare
OR: Odds ratio
PMTCT: Prevention of mother to child transmission
PTB: Pulmonary TB
SARS-CoV-2: Severe acute respiratory syndrome coronavirus 2
TB: Tuberculosis
US, USA: United States of America
WHO: World Health Organization
Xpert: Xpert® MTB/RIF test for detecting Mycobacterium tuberculosis (MTB) and rifampin (RIF) resistance (Cepheid, Sunnyvale, California, USA)
