# Supplementary material for: HIV matters when diagnosing TB in young children: an ancillary analysis in children enrolled in the INPUT stepped wedge cluster randomized study
Source: BMC Infect Dis. 2023 Apr 17;23:234. doi: 10.1186/s12879-023-08216-w (PMC10107571; doi:10.1186/s12879-023-08216-w)
Supplement: Supplementary file 1 — Supplementary Material 1 [file 12879_2023_8216_MOESM1_ESM.pptx]

## Slide 1
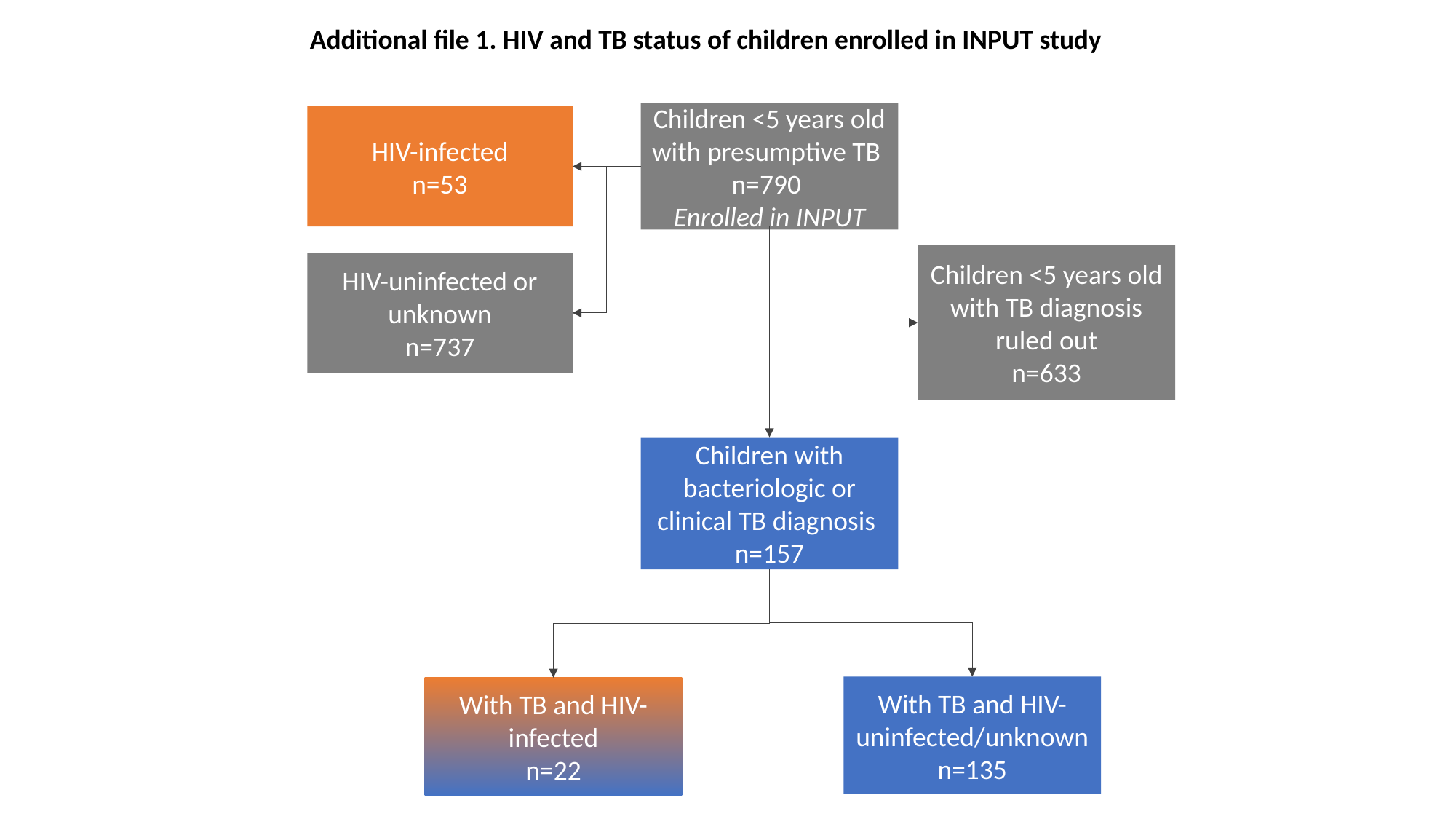

Additional file 1. HIV and TB status of children enrolled in INPUT study
Children <5 years old with presumptive TB
n=790
Enrolled in INPUT
HIV-infected
n=53
Children <5 years old with TB diagnosis ruled out
n=633
HIV-uninfected or unknown
n=737
Children with bacteriologic or clinical TB diagnosis
n=157
With TB and HIV-uninfected/unknown
n=135
With TB and HIV-infected
n=22
